# Supplementary material for: Improving social determinants of health documentation in French electronic health records using large language models
Source: Sci Rep. 2025 Nov 26;15:45427. doi: 10.1038/s41598-025-29987-z (PMC12749410; doi:10.1038/s41598-025-29987-z)
Supplement: Supplementary file 1 — Supplementary Information. [file 41598_2025_29987_MOESM1_ESM.docx]

**SUPPLEMENTAL MATERIAL**

| **SDoH categories** | **Z-codes** |
| --- | --- |
| Alcohol (current, past) | Z502 (alcohol withdrawal), Z714 (advice and monitoring for alcoholism) |
| Tobacco (current) | Z720 (smoking-related difficulties) |
| Drug | Z503 (rehabilitation for drug addicts and after drug abuse) |
| Living condition (alone) | Z290 (isolation), Z602 (difficulties linked to loneliness) |
| Descendants (yes) | Z370 (single birth, living child), Z372 (twin birth, twins born alive), Z391 (breastfeeding care and examinations), Z392 (routine postpartum check-up) |
| Housing (no) | Z590 (difficulties associated with homelessness), Z598 (other difficulties related to housing and economic conditions) |
| Ethnicity/Country of birth | Z603 (acculturation difficulties) |

Supplementary Table 1. Z-codes mapping to SDoH categories

| **Error Type** | **Count** |
| --- | --- |
| Human annotation errors | 105 |
| False negatives | 224 |
| False positives | 125 |
| Output not structured as specified in the model training | 63 |
| Incorrect labels | 58 |
| Postprocessing error (multiple matches in the source text) | 26 |
| Correct label but different text spans | 94 |
| Tokenization-related errors | 3 |

Supplementary Table 2. Most common types of discrepancies between ground truth and model predictions.

| Entity | Relation | Attribute | MUSCADET-InHouse  (n=1,700) | | MUSCADET-Synthetic  (n=340) | | UWFrenchSDOH  (n=364) | | InHouse Tuberculosis and ALS (n=400) | |
| --- | --- | --- | --- | --- | --- | --- | --- | --- | --- | --- |
|  |  |  | i | n | i | n | i | n | i | n |
| Alcohol | Amount | - | 209 | 189 | 36 | 36 | 26 | 26 | 6 | 6 |
| Alcohol | Duration | - | 5 | 5 | 2 | 2 | 3 | 3 | 2 | 2 |
| Alcohol | Frequency | - | 358 | 336 | 56 | 56 | 63 | 62 | 11 | 10 |
| Alcohol | History | - | 53 | 53 | 5 | 5 | 5 | 5 | 3 | 3 |
| Alcohol | StatusTime | current | 342 | 340 | 67 | 66 | 78 | 78 | 20 | 13 |
| Alcohol | StatusTime | none | 278 | 278 | 110 | 110 | 155 | 155 | 17 | 17 |
| Alcohol | StatusTime | past | 77 | 76 | 17 | 17 | 17 | 16 | 5 | 4 |
| Alcohol | Type | - | 141 | 113 | 37 | 36 | 8 | 8 | 5 | 5 |
| Descendants_No | - | - | 136 | 136 | 33 | 33 | 5 | 5 | 6 | 6 |
| Descendants_Yes | Amount | - | 998 | 845 | 136 | 117 | 57 | 49 | 46 | 40 |
| Descendants_Yes | Type | - | 1131 | 901 | 150 | 128 | 80 | 64 | 59 | 48 |
| Descendants_Yes | - | - | 38 | 34 | 3 | 3 | 0 | 0 | 5 | 5 |
| Drug | Amount | - | 12 | 11 | 13 | 11 | 0 | 0 | 2 | 2 |
| Drug | Duration | - | 1 | 1 | 1 | 1 | 0 | 0 | 1 | 1 |
| Drug | Frequency | - | 16 | 16 | 16 | 13 | 1 | 1 | 6 | 5 |
| Drug | History | - | 7 | 6 | 0 | 0 | 10 | 5 | 2 | 2 |
| Drug | StatusTime | current | 26 | 25 | 30 | 26 | 5 | 4 | 8 | 6 |
| Drug | StatusTime | none | 73 | 71 | 65 | 65 | 114 | 113 | 4 | 4 |
| Drug | StatusTime | past | 11 | 9 | 6 | 6 | 17 | 12 | 3 | 3 |
| Drug | Type | - | 45 | 35 | 31 | 23 | 23 | 10 | 12 | 8 |
| Education | History | - | 1 | 1 | 0 | 0 | 0 | 0 | 0 | 0 |
| Education | - | - | 80 | 75 | 20 | 20 | 3 | 3 | 1 | 1 |
| Employment_Other | History | - | 21 | 20 | 0 | 0 | 2 | 2 | 0 | 0 |
| Employment_Other | - | - | 96 | 95 | 21 | 21 | 14 | 13 | 8 | 8 |
| Employment_Pensioner | History |  | 24 | 24 | 4 | 4 | 2 | 2 | 1 | 1 |
| Employment_Pensioner | - | - | 417 | 404 | 44 | 44 | 32 | 32 | 22 | 19 |
| Employment_Student | - | - | 48 | 44 | 17 | 17 | 8 | 8 | 0 | 0 |
| Employment_Unemployed | History | - | 8 | 8 | 0 | 0 | 0 | 0 | 0 | 0 |
| Employment_Unemployed | - | - | 154 | 150 | 17 | 16 | 9 | 9 | 7 | 7 |
| Employment_Working | - | - | 493 | 475 | 114 | 111 | 56 | 54 | 15 | 13 |
| Ethnicity |  | -- | 103 | 102 | 18 | 18 | 1 | 1 | 27 | 25 |
| Housing_No | - | - | 22 | 17 | 13 | 13 | 0 | 0 | 2 | 2 |
| Housing_Yes | History |  | 3 | 3 | 0 | 0 | 0 | 0 | 0 | 0 |
| Housing_Yes | - | - | 968 | 812 | 122 | 115 | 48 | 43 | 137 | 70 |
| Income | History | - | 1 | 1 | 0 | 0 | 0 | 0 | 0 | 0 |
| Income | - | - | 39 | 34 | 8 | 8 | 0 | 0 | 2 | 2 |
| Job | - | - | 1184 | 1060 | 216 | 204 | 95 | 86 | 58 | 44 |
| Last_job |  | -- | 1068 | 1034 | 204 | 204 | 85 | 85 | 49 | 44 |
| Living_Alone | - | - | 281 | 277 | 37 | 37 | 17 | 17 | 10 | 10 |
| Living_WithOthers | History | - | 4 | 4 | 0 | 0 | 0 | 0 | 0 | 0 |
| Living_WithOthers |  | -- | 597 | 593 | 89 | 89 | 73 | 73 | 44 | 43 |
| MaritalStatus_Divorced | History | - | 5 | 5 | 0 | 0 | 1 | 1 | 0 | 0 |
| MaritalStatus_Divorced | - | - | 92 | 86 | 17 | 17 | 17 | 16 | 8 | 7 |
| MaritalStatus_InRelationship | Duration | - | 0 | 0 | 1 | 1 | 1 | 1 | 0 | 0 |
| MaritalStatus_InRelationship | History | - | 3 | 3 | 0 | 0 | 0 | 0 | 0 | 0 |
| MaritalStatus_InRelationship | - | - | 882 | 774 | 131 | 125 | 126 | 101 | 72 | 44 |
| MaritalStatus_Single | - | - | 73 | 73 | 20 | 20 | 20 | 20 | 6 | 6 |
| MaritalStatus_Widowed | History | - | 21 | 21 | 2 | 2 | 2 | 2 | 0 | 0 |
| MaritalStatus_Widowed | - | - | 73 | 72 | 16 | 16 | 7 | 7 | 3 | 3 |
| PhysicalActivity_No | History | - | 1 | 1 | 0 | 0 | 0 | 0 | 0 | 0 |
| PhysicalActivity_No |  | -- | 53 | 52 | 14 | 14 | 3 | 3 | 0 | 0 |
| PhysicalActivity_Yes | Amount | - | 34 | 29 | 5 | 5 | 6 | 4 | 1 | 1 |
| PhysicalActivity_Yes | Duration | - | 2 | 2 | 2 | 2 | 0 | 0 | 0 | 0 |
| PhysicalActivity_Yes | Frequency | - | 47 | 37 | 11 | 9 | 8 | 5 | 1 | 1 |
| PhysicalActivity_Yes | Type | - | 0 | 0 | 0 | 0 | 0 | 0 | 1 | 1 |
| PhysicalActivity_Yes | - | - | 163 | 138 | 52 | 43 | 13 | 7 | 2 | 2 |
| Tobacco | Amount | - | 385 | 313 | 63 | 60 | 59 | 59 | 27 | 22 |
| Tobacco | Duration | - | 67 | 66 | 6 | 6 | 29 | 29 | 7 | 7 |
| Tobacco | Frequency | - | 260 | 244 | 41 | 40 | 63 | 62 | 17 | 16 |
| Tobacco | History | - | 213 | 212 | 33 | 32 | 33 | 32 | 7 | 7 |
| Tobacco | StatusTime | current | 247 | 243 | 67 | 66 | 47 | 46 | 28 | 21 |
| Tobacco | StatusTime | none | 407 | 403 | 104 | 104 | 159 | 159 | 18 | 18 |
| Tobacco | StatusTime | past | 237 | 235 | 48 | 48 | 54 | 52 | 14 | 13 |
| Tobacco | Type | - | 213 | 202 | 43 | 42 | 43 | 43 | 19 | 17 |

Supplementary Table 3. Distribution of all entity-relation-attribute combinations across all datasets. The i denotes the number of instances of a given combination, and n denotes the number of documents in which at least one occurrence of the combination appears.
